# Neuroimmune interactions in arthritis: linking pain sensitisation and inflammation

**DOI:** 10.1007/s00774-025-01678-9

**Published:** 2025-12-27

**Authors:** Tammie Tao Min Sow, Tetsuo Hasegawa

**Affiliations:** Molecular Immunity Unit, Department of Medicine, Medical Research Council Laboratory of Molecular Biology, University of Cambridge, Cambridge, CB2 0QH UK

**Keywords:** Neuroimmune interaction, Synovium, Nociceptor, Macrophage, Arthritis

## Abstract

Arthritis represents a group of chronic joint diseases characterised by persistent inflammation, pain, and progressive tissue damage. Despite advances in therapeutic management, many patients experience incomplete symptom relief, highlighting the need to better understand the underlying mechanisms that sustain inflammation and pain. Emerging evidence indicates that neuroimmune interactions within the joint microenvironment play a central role in the pathogenesis of arthritis. The synovium, a thin membrane that lines the joint cavity, is the primary site of pathology in arthritis and serves as a dynamic interface integrating immune, vascular, and neural components. Under physiological conditions, tissue-resident macrophages, fibroblasts, and sensory nerve fibres maintain joint homeostasis. However, during arthritis, the synovium undergoes extensive remodelling, including hyperplasia, angiogenesis, and nerve fibre sprouting, which together amplify inflammatory and nociceptive signalling. Distinct macrophage subsets within the synovium exhibit specialised roles in mediating inflammation and communicating with neurons. Macrophage-derived cytokines such as IL-1β, IL-6, and TNF-α can directly sensitise nociceptors, whilst chemokines like CCL2 engage neuronal receptors to enhance excitability. Conversely, activated sensory neurons release neuropeptides such as calcitonin gene-related peptide (CGRP) and substance P (SP), which can modulate immune cell behaviour. Sympathetic signalling further contributes to immune modulation and correlates with disease severity. Together, these studies reveal that arthritis progression and chronic pain are shaped by reciprocal signalling between the nervous and immune systems. Understanding these complex pathways offers new perspectives for therapeutic intervention, suggesting that targeting neuroimmune crosstalk could provide dual benefits—reducing inflammation whilst alleviating chronic pain in arthritic disease.

## Introduction

C. Arthritis encompasses a group of joint diseases characterised by inflammation, which may be acute or chronic. The most common forms of arthritis in adults are rheumatoid arthritis (RA) and osteoarthritis (OA), both collectively affecting millions worldwide [1, 2]. Characterised by chronic pain and progressive tissue damage in the joint, these conditions impose substantial health and socioeconomic burdens. There is increasing prevalence of these diseases due to ageing and obesity, and women are more affected by arthritis than men due to sex-related differences [3]. OA is one of the most common forms of arthritis in adults and is typically a result of overusing or injury of the joint, causing tissue damage [4]. OA is characterised by cartilage loss and subchondral bone lesions, which lead to osteophyte formation and accumulation of macrophages in the intimal lining [4]. RA is an autoimmune disease affecting the joint, and the disease manifests in the form of heightened inflammatory state and the presence of autoantibodies, which eventually leads to tissue damage [5].

Chronic pain is one of the significant hallmarks in arthritis. Increasing evidence suggests that pain and inflammation are closely linked through neuroimmune interactions within the joint microenvironment [6]. Amongst the immune cells in the joint, macrophages are the key players in neuroimmune crosstalk. In response to damage-associated molecular patterns (DAMPs) from tissue damage in the context of OA, they can secrete inflammatory mediators such as cytokines, chemokines, and growth factors under arthritic conditions. RA joints, on the other hand, have elevated levels of cytokines and chemokines due to a pro-inflammatory state induced by autoantibodies. Emerging evidence has shown that these molecules can induce sensitisation of sensory neurons, thus eliciting a pain response [7]. Additionally, sensory neurons known as nociceptors can release neuropeptides in response to noxious stimuli, and recent evidence has shown that immune cells express the receptors for these neuropeptides, thus enabling them to elicit a response [7]. Understanding these interactions has become essential for identifying new therapeutic strategies that address not only structural damage but also persistent pain in arthritis.

In this review, we summarise the latest information about the joint microenvironment under healthy and disease conditions and discuss recent findings that could suggest neuroimmune crosstalk possibly contributing to arthritis in synovial joints.

## The healthy synovium and joint microenvironment

The core structure of the joint microenvironment is the synovium, which is the thin membrane that encapsulates the fluid-filled joint cavity [8]. Its main function includes lubricating the joint to minimise friction and providing metabolic and nutritional support to the synovial cavity [8]. The synovial membrane is formed by two layers, the lining layer adjacent to the joint cavity, and the sublining layer [8]. Under steady state, the lining layer is 1–2 cells thick and is composed of tissue resident macrophages and fibroblasts [8]. The sublining layer also contains tissue macrophages and fibroblasts, but in addition, it also contains blood and lymphatic vessels, nerve fibres, and adipose cells [8].

In the healthy joint, vascular and neuronal networks maintain homeostasis by regulating nutrient delivery, fluid exchange, and sensory input. Synovial joints are innervated by myelinated Aδ-fibres and unmyelinated peptide-rich C-fibres known as nociceptors [9]. These fibres have a high stimulation threshold and can respond to mechanical, thermal, and chemical stimuli [7, 10]. They are essential for detecting noxious stimuli and maintaining joint homeostasis, and they form a crucial link between neural and immune signalling. Na_V_1.8 is a voltage-gated ion channel commonly used to identify sensory neurons and comprises approximately 75% of dorsal root ganglion (DRG) sensory neurons, including > 90% of C-nociceptors, as well as a small fraction of Aδ-nociceptors and Aβ afferents [11]. In a knee joint of 10-week-old Na_V_1.8-tdTomato reporter mice, there was high innervation of Na_V_1.8^+^ fibres at the lateral synovium [11]. The most known ligand-gated ion channel in nociceptors is transient receptor potential vanilloid 1 (TRPV1) [7]. Upon sensing a stimulus, TRPV1 allows influx of cations and depolarises the membrane, creating an action potential that is relayed to the spinal cord [7]. These peptidergic nociceptors then relay the response signal from the cell body to the periphery by releasing neuropeptides like calcitonin-gene related peptide (CGRP) and Substance P (SP) [7, 10]. Sympathetic nerve fibres can also be found in the synovial joints near blood vessels, and these fibres are tyrosine hydroxylase (TH)-positive [12]. Imaging studies found that sympathetic nerve fibres are closely associated with αSMA^+^ arterioles, which justifies their function in secreting neurotransmitters associated with vasoregulation like neuropeptide Y (NPY), noradrenaline (NA), and vasoactive intestinal peptide (VIP) [12]. These spatial relationships suggest that neural and vascular components can directly influence immune activation and inflammation in arthritis.

In addition to neural innervation, the synovium’s vascular network plays a pivotal role in regulating nutrient exchange and immune accessibility. Previously, the gold standard for joint imaging was through sagittal sections of the joint; however, this only provides a limited snapshot of the tissue. The emergence of new imaging techniques allowed further understanding of the vasculature and immune cell composition in the synovium. With the whole mount synovium imaging system, which enables visualisation of the entire synovium in three dimensions, we have a better understanding of the location of PV1^+^ fenestrated capillaries within the mouse synovium [13]. They were located at the lining–sublining (L–SL) interface and near the synovial–bone interface at the periphery of the joint [13]. Furthermore, CGRP^+^ nociceptor neuronal fibres were found branching into the L–SL interface around PV1^+^ capillaries [13]. These fenestrated capillaries were also found in human synovium, and their location aligns with regions in the joint that are most susceptible to inflammatory granulomatous lesions in arthritis, called pannus [13, 14]. When assessed for their function in vivo, they have been found to allow circulating stimuli to extravasate into the joint microenvironment, further suggesting that factors causing arthritis can gain access to the joint through these fenestrated capillaries [13]. This may also explain why a variety of systemic disease models, such as PD1-knock out mice [15], MRL/lpr mice [16], or transgenic mice expressing hTNF [17], are accompanied by arthritis.

**Fig. 1:**
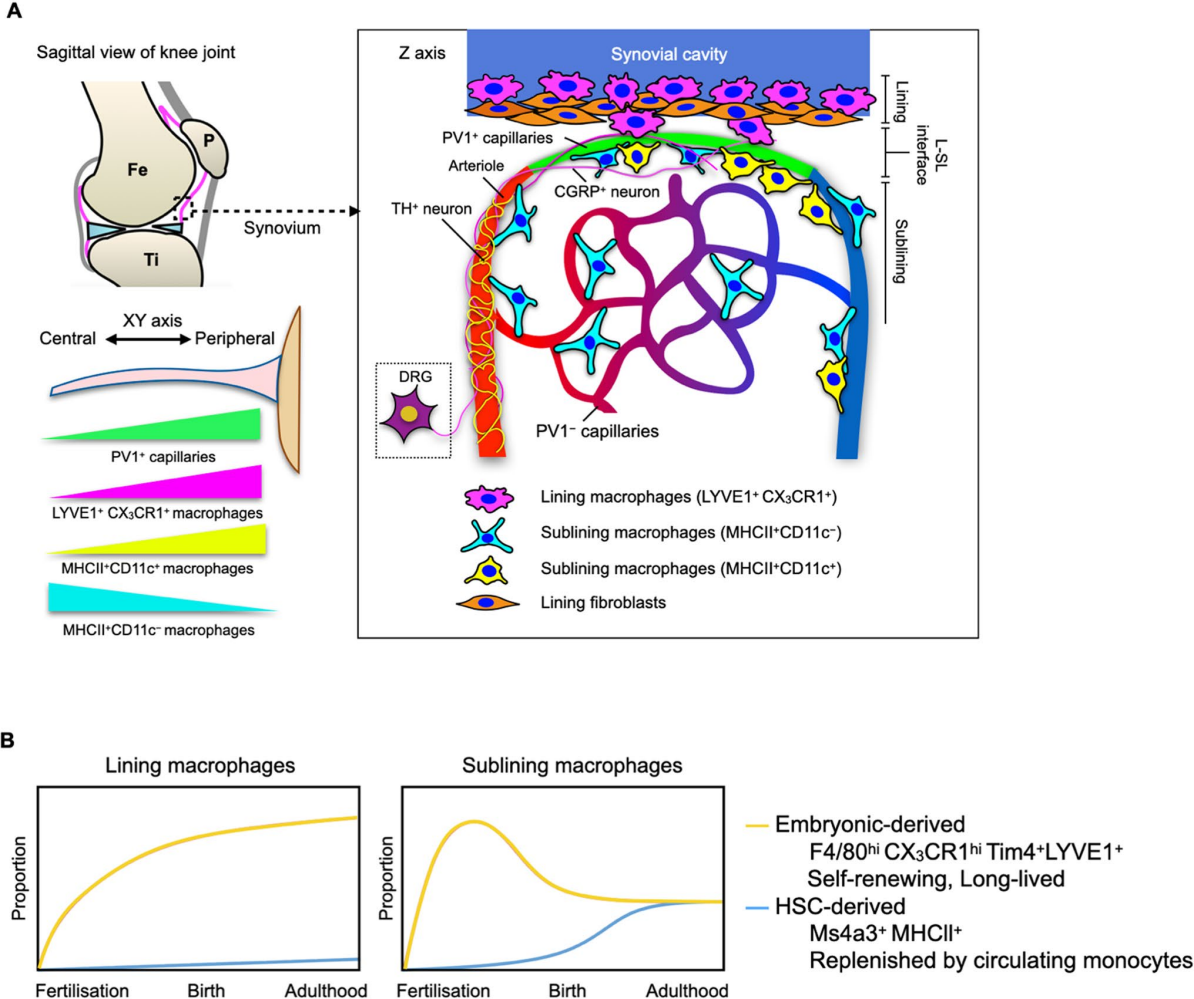
The synovium and the ontogeny of synovial tissue macrophages. **A** Top left diagram shows the sagittal view of the knee joint and the synovial membrane is depicted in magenta. Bottom left diagram shows the density of fenestrated capillaries and different macrophage populations from central point to the periphery of the joint. Diagram on right shows the distribution of the different macrophage and nerve fibres in the joint, with lining layer formed by LYVE1^+^CX3CR1^+^ macrophages (magenta), with supporting sublayer of lining fibroblasts (orange). MHCII^+^CD11c^−^ (cyan) and MHCII^+^CD11c^+^ (yellow) macrophages survey the area around PV1^+^fenestrated capillaries. TH^+^ neurons are closely associated with the arterioles and CGRP^+^ near lateral synovium. **B** Primitive macrophages (F4/80pos, CD11bneg) contribute to most of the lining and sublining layer STM during gestation. After birth, there are increasing contributions of monocyte-derived CD11bpos macrophages to the sublining layer

Macrophages and fibroblasts are present in both the lining and sublining layers of the synovium [8]. Synovial fibroblasts are thought to be the main source of hyaluronan in synovial fluid, and they prominently express adhesion molecules including vascular cell adhesion molecule-1 (VCAM-1), intercellular adhesion molecule (ICAM)-1, CD44, and β_1_ integrins [8]. Exposure of fibroblasts to immune mediators and cytokines can drive them into an inflammatory-like state [18]. Synovial macrophages are thought to be distinct from monocyte-derived macrophages. These macrophages are found to be transcriptionally distinct, with the lining macrophages being LYVE1^+^CX_3_C-chemokine receptor 1-positive (CX_3_CR1^+^), and interstitial macrophages being CX_3_CR1^−^ [19]. The CX_3_CR1^−^ macrophage population can also locally proliferate and maintain the pool of LYVE1^+^CX_3_CR1^+^ lining macrophages independent of blood monocytes [19, 20]. Within the CX_3_CR1^−^ population, unsupervised assessment of myeloid markers showed that they can be further divided into two subclusters—major histocompatibility complex class II^+^CD11c^–^ (MHCII^+^CD11c^–^) and MHCII^+^CD11c^+^ populations [13]. Utilising the Ms4a3^Cre^-Rosa^tdTomato^ model, it was shown that nearly 50% of MHC-II^+^ macrophages were tdTomato^+^, indicating a bone marrow-derived origin. In contrast, the majority of lining layer LYVE1^+^CX_3_CR1^+^ macrophages were tdTomato^−^; consistent with an embryonic origin [13, 20]. When studying the spatial information of the three macrophage clusters in the joint, it was found that MHCII^+^CD11c^+^ macrophages were most closely associated with PV1^+^ capillaries, whereas the MHCII^+^CD11c^−^ macrophage cluster was distributed near both PV1^−^ and PV1^+^ capillaries [13]. This spatial organisation implies that macrophage subsets positioned near vascular structures may serve as key intermediaries in neurovascular and immune communication within the joint (Fig. 1).

In summary, the joint microenvironment is an intricate tissue that integrates mechanical, immune, and neural signals to preserve joint integrity and function. The anatomical proximity between nerve fibres, capillaries, and immune cells establishes the synovium as a key interface for neuroimmune communication, namely the blood-joint barrier (BJB) and a focal point for studying arthritis pathogenesis.

## Changes in joint homeostasis and neuroimmune interactions during arthritis

**Fig. 2:**
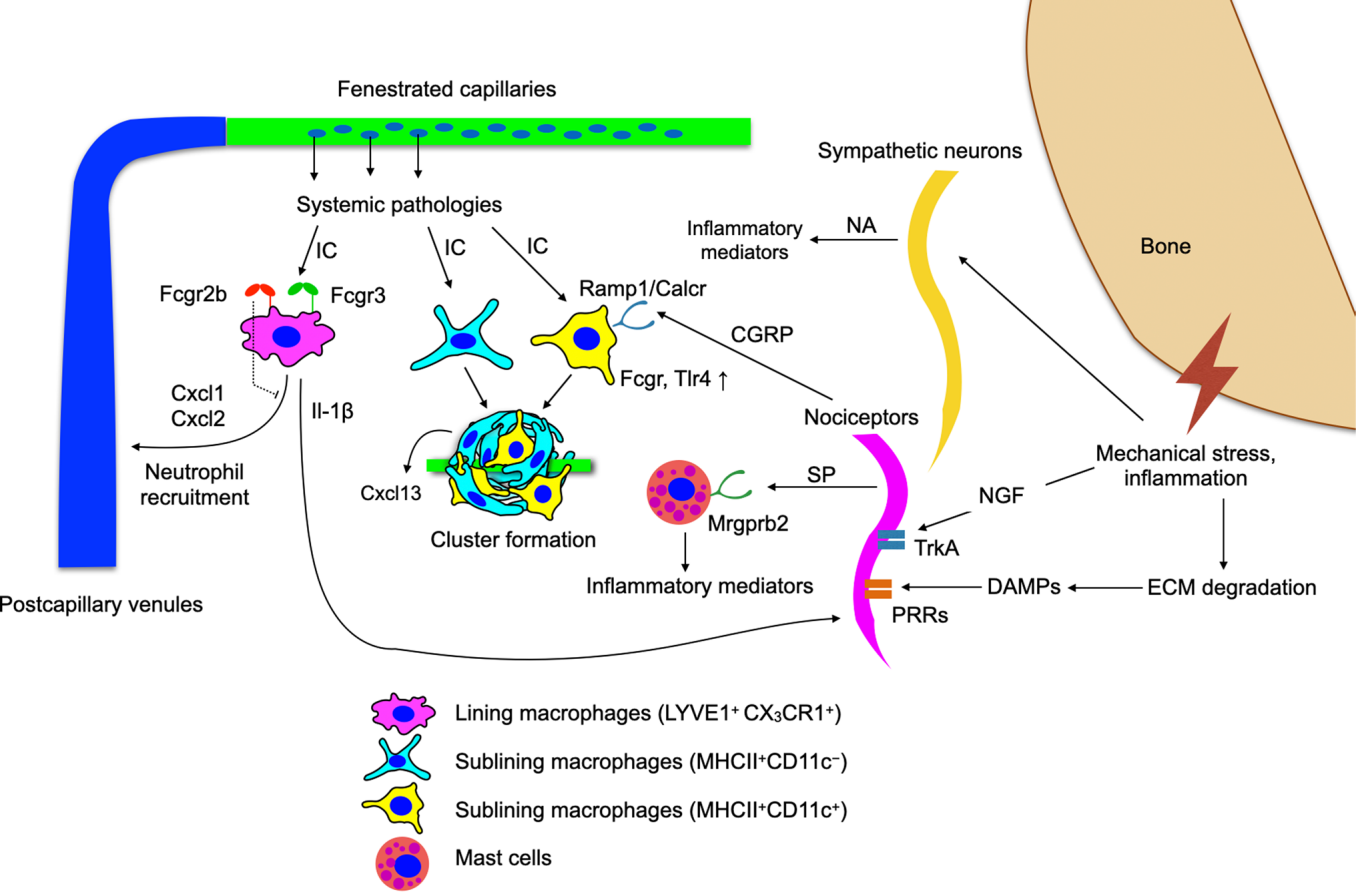
Neuroimmune interactions in the joint under arthritic conditions caused by bone damage (in OA) and systemic pathologies infiltrating into the joint. DAMPs and NGF can be released because of OA, and this can cause activation of nociceptors and sympathetic neurons. This can then lead to downstream signalling to immune cells, particularly the MHCII^+^CD11c^+^ (yellow) macrophages. Systemic pathologies can also affect the macrophages in the joint, and lining macrophages (LYVE1^+^CX_3_CR1^+^) can signal to the nociceptors via IL-1ß, and this can create a positive feedback loop

The synovium undergoes profound structural and cellular changes during arthritis, including synovial lining hyperplasia, angiogenesis, and nerve fibre remodelling. These changes collectively amplify inflammation and pain signalling within the joint. In this section, we will discuss the various interactions that occur between the neural and immune compartment (Fig. 2; Table 1).

**Table 1:** Neuroimmune interactions and the potentially relevant diseases

Direction of interaction	Mechanism	Relevant diseases	References
Immune to neuron	Lining macrophages activate nociceptors through IL-1ß upon IC challenge	RA, SLE	[13]
	NGF induces ectopic sprouting of neuro-fibres	RA, OA	[23]
	NGF enhances nociceptive sensitisation by increasing TRPV1 expression	RA, OA	[26]
	CCL2 increases Na_v_1.8 and TRPV1 sodium channel activity	RA, OA	[29, 30]
	DAMPs excite nociceptors through TLR4 signalling	OA	[32]
	TNF-α upregulates Na_v_1.8 in DRG neurons	RA, OA	[34]
	gp130 contributes to joint pain	RA	[35]
Neuron to immune	CGRP from nociceptors enhance macrophage response via upregulating TLR4 and FcgR	RA, SLE	[13]
	CGRP promotes a regulatory phenotype in TLR4‐stimulated macrophages	RA, SLE	[38]
	Substance P enhances NF-kB transactivation and chemokine response in murine macrophages	RA, SLE	[40]
	NPY upregulates inflammatory cytokines	RA	(44)

*RA* Rheumatoid arthritis, *OA* osteoarthritis, *SLE* systemic lupus erythematosus

In a recent study, it was shown that macrophages have subset-specific functions in response to joint inflammation [13]. IC (ovalbumin (OVA) opsonized with a polyclonal anti-OVA IgG) challenge is one of the common models used to study diseases caused by type III hypersensitivity, such as SLE and reactive arthritis. In an IC-challenged mouse model, it was found that all three macrophage subsets could internalise the ICs, but each had differences in their upregulated genes [13]. The MHCII^+^CD11c^+^ macrophages had an upregulation of antigen processing and presentation related gene sets, whereas the LYVE1^+^CX_3_CR1^+^ macrophages were found to have upregulated leukocyte activation and neutrophil recruitment gene sets [13]. This was also supported by an increase of CXCL1 and CXCL2 production, both of which are prominent chemokines for neutrophil recruitment [13, 21]. Enrichment analysis also showed that there was an increase in chemokine expression and interferon and interleukin signalling pathways in fibroblasts, all of which are inflammatory pathways [13]. When anti-CSF1R was used to deplete LYVE1^+^ macrophages in the joint, it was found that this attenuated CGRP^+^ nociceptor activation, further indicating that the macrophages are key players in neuroimmune interactions [13].

The change in innervation pattern of nerve fibres under arthritis is still inconclusive, though some studies seem to show an increase in neuron fibre density in the synovium in arthritis mouse models [22, 23]. Nerve growth factor (NGF) is an essential neurotrophin for the development and survival of nerve fibres, and it can be produced at substantial amounts at inflammatory sites [24]. Tyrosine kinase receptor A (TrkA) is the receptor for NGF, and it was found that there is strong expression of TrkA on nociceptors, accounting for the strong effect of NGF on these nerve fibres [25]. Upon binding to NGF, it can enhance TRPV1 and Na_v_1.8 signalling in nociceptors [25, 26]. This induces spontaneous discharge of nociceptors and increases the production of CGRP and SP [24], lowering the threshold for eliciting pain signals.

Besides NGF, there are many other molecules that act as inflammatory mediators, such as DAMPs due to tissue damage, prostaglandin E2, bradykinin, cytokines, and chemokines [27]. Healthy synovium typically does not have high immune cell trafficking, but under increased cytokine and chemokine expression in OA and RA joints, the infiltration of immune cells also increases [28]. CCR2, a receptor for the chemokine CCL2, is found to be expressed in sensory neurons, and their activation was shown to sensitise nociceptors through the increase in Na_v_1.8 and TRPV1 expression by the activation of the PI3K/Akt signalling pathway [29, 30]. Some evidence also shows that CCL2 can be expressed by DRG neurons themselves, which suggests that pain transmission can happen in an autocrine manner [30].

Extravasation of immune cells by attraction of chemokines can then lead to high levels of pro-inflammatory cytokines in the joint microenvironment. Pro-inflammatory cytokines can activate pattern recognition receptors (PRRs), and toll-like receptors (TLRs) amongst all have been characterised to the greatest extent and shown to be involved in chronic pain [31]. Studies have shown that TLR4 is expressed on nociceptors and that they can be activated directly by DAMPs such as S100A8 and α_2_-macroglobulin, thus linking inflammation to pain sensitisation in arthritis [32]. However, deletion of TLR4 alone was not sufficient to suppress mechanical allodynia, suggesting there are other PRRs that can play a role in nociceptor sensitisation [32].

Moreover, a proportion of nociceptors were also found to express the receptors for pro-inflammatory cytokines, thus implying that cytokines mediate pain by directly acting on the nociceptors [33]. Injection of TNF-α has been shown to increase NGF production and a progressive increase of nociceptor responses by upregulating the expression of Na_v_1.3 and Na_v_1.8 [34]. Nociceptors also express gp80 and gp130, which are subunits of the IL-6 receptor [33]. Mechanical hyperalgesia was found to be reduced when IL-6/IL-6R complexes were neutralised in an AIA-arthritis rat model, further proving that IL-6 can lead to the induction of pain signals [35].

Nociceptors are also sensitive to the proinflammatory cytokine interleukin-1-beta by acting in a p38 mitogen-activated protein kinase (p38 MAP kinase)-dependent manner (IL-1ß) [27]. In a recent study, *Cx3cr1*^CreER^*Il-1β*^fl/fl^ mice were used to study the effects of depleting synovial macrophage-derived IL-1ß on nociceptor activation [13]. Consistent with previous findings, nociceptor activation was reduced by measuring CGRP expression, and this effect was seen specifically in L4 DRG, where joint-innervating neuron cell bodies reside [13]. This further shows that macrophages in the joint are a huge point of communication between the neural and immune compartments, and IL-1ß is the key molecule involved in this process.

In patients with RA, antibodies reactive to self-antigens (known as autoantibodies) are present in the joint microenvironment. These antibodies can further increase the inflammatory responses through activation of the complement system or binding of Fc gamma receptors, which can indirectly activate nociceptors [36]. Together, these immune-derived mediators act on sensory nerve terminals, creating a pro-nociceptive microenvironment that sustains chronic pain.

Neuroimmune interactions are bidirectional; hence, they can also occur via the release of neuropeptides by activated nerve fibres and their effect on immune cells. The most common neuropeptides released by nociceptors are CGRP and SP. The receptor for CGRP is formed by receptor activity modifying protein 1 (*Ramp1*) and its co-receptor, calcitonin receptor-like receptor (*Calcrl*) [37]*.* It was found recently that MHCII^+^CD11c^+^ synovial macrophages express *Ramp1* [13]. Earlier studies seem to imply that CGRP induces a regulatory (M2) phenotype in TLR4-activated macrophages in vitro and were validated by the production of IL-10 [38]. CGRP stimulation of whole synovial explants resulted in upregulation of *Fcgr2b* and *Fcgr3* in MHCII^+^CD11c^+^ macrophages, which enhance their responses upon binding to the Fc region of IgG [13]. In addition to that, it was noted that there was upregulation of *Tlr4* in immune cells upon CGRP stimulation, which could lead to downstream cytokine signalling. This suggests that CGRP signalling in the synovium exerts complex and context-dependent effects on macrophages, balancing anti-inflammatory and pro-inflammatory pathways that ultimately influence joint inflammation and pain.

SP is also a prominent neuropeptide released by nociceptors in response to noxious stimuli [39]. When murine macrophages were stimulated with SP, it was shown that it induced NF-κB activation, which is a common transcription factor for pro-inflammatory cytokine production [40]. Neurokinin (NK)-1 is the receptor for SP binding, and it was also shown that when the NK-1 antagonist was administered, there was a reduction in the production of chemokines like macrophage inflammatory protein-2 (MIP-2) and monocyte chemoattractant protein-1 (MCP-1) [40]. This implies that SP has a pro-inflammatory role. Since pro-inflammatory cytokines such as TNF-α and IL-1β can also upregulate neuropeptide release in nociceptors, as mentioned earlier, this suggests a positive feedback loop that perpetuates neurogenic inflammation and pain.

In a study involving co-culture of nociceptors with dendritic cells (DCs), it was found that the presence of nociceptors can enhance IL-12 production in DCs [41]. However, it was noted that the interaction between them occurs not through neuropeptide signalling but through physical contact [41]. Although this was not studied in the joint, this could imply that the presence of nociceptors can induce a more pro-inflammatory state in immune cells that cannot be achieved by mere neuropeptide co-stimulation.

Beyond sensory neurons, sympathetic signalling also shapes inflammation in the joint. NPY is co-released with NA and upon sympathetic neuron stimulation [42], and immune cells were found to express different variations of the NPY receptors, suggesting they are responsive to NPY signalling [43]. In a study utilising rapamycin to reduce the incidence of arthritis, NPY expression was found positively correlated with arthritis severity [44]. *Npy* knockdown resulted in significantly reduced expression of the cytokines *Tnfa*, *Il1b*, and *Il6 *[44], further suggesting that there is crosstalk between the sympathetic nervous compartment and immune cells.

## Conclusion

Collectively, these findings illustrate a tightly interwoven network of communication between neurons and immune cells in the arthritic joint. Through reciprocal release of cytokines, chemokines, and neuropeptides, both systems amplify inflammation and pain signalling. However, some of the findings need to be verified in the joint microenvironment, and further studies need to be carried out to understand the mechanistic properties of these interactions. Understanding these bidirectional pathways provides insight into potential therapeutic targets aimed at disrupting neuroimmune interactions in arthritis.
